# Semantic-Aware Remote Sensing Change Detection with Multi-Scale Cross-Attention

**DOI:** 10.3390/s25092813

**Published:** 2025-04-29

**Authors:** Xingjian Zheng, Xin Lin, Linbo Qing, Xianfeng Ou

**Affiliations:** 1 College of Design and Engineering, National University of Singapore, Singapore 119077, Singapore; e0679973@u.nus.edu; 2 School of Information Science and Engineering, Hunan Institute of Science and Technology, Yueyang 414000, China; 14203101995@vip.hnist.edu.cn; 3 College of Electronics and Information Engineering, Sichuan University, Chengdu 610065, China; qing_lb@scu.edu.cn

**Keywords:** change detection (CD), convolutional neural network (CNN), deep learning (DL), transformer, semantic map

## Abstract

Remote sensing image change detection plays a vital role in diverse real-world applications such as urban development monitoring, disaster assessment, and land use analysis. As deep learning strives, Convolutional Neural Networks (CNNs) have shown their effects in image processing applications. There are two problems in old-school change detection techniques: First, the techniques do not fully use the effective information of the global and local features, which causes their semantic comprehension to be less accurate. Second, old-school methods usually simply rely on differences and computation at the pixel level without giving enough attention to the information at the semantic level. To address these problems, we propose a multi-scale cross-attention network (MSCANet) based on a CNN in this paper. First, a multi-scale feature extraction strategy is employed to capture and fuse image information across different spatial resolutions. Second, a cross-attention module is introduced to enhance the model’s ability to comprehend semantic-level changes between bitemporal images. Compared to the existing methods, our approach better integrates spatial and semantic features across scales, leading to more accurate and coherent change detection. Experiments on three public datasets (LEVIR-CD, CDD, and SYSU-CD) demonstrate competitive performance. For example, the model achieves an F1-score of 96.19% and an IoU of 92.67% on the CDD dataset. Additionally, robustness tests with Gaussian noise show that the model maintains high accuracy under input degradation, highlighting its potential for real-world applications. These findings suggest that our MSCANet effectively improves semantic awareness and robustness, offering a promising solution for change detection in complex and noisy remote sensing environments.

## 1. Introduction

Remote sensing image change detection (RSICD) is an essential technique that researchers use to analyze differences among images of the same area taken at different times, which has been playing a role in environmental monitoring, urban planning, agricultural management, and disaster response. By comparing images taken at different times, change detection can reveal dynamic changes on the ground surface and land use, giving people valuable information to understand environmental changes.

In the early stage of the change detection field, due to the weak points of sensor and satellite technology, the resolution of remote sensing images was low, so the change detection techniques were relatively simple. People first performed basic algebraic operations on image pixel pairs to produce difference maps and then set threshold values to classify image changes. Gillespie et al. [1] used the Euclidean Distance (ED) as the calculation method to obtain the magnitude metric of change detection. Celik [2] proposed a method for multi-temporal remote sensing images, which distinguishes changed pixels from unchanged pixels by dividing the feature vectors of pixel pairs into two clusters. Nielsen et al. used the Multivariate Alteration Detection (MAD) method [3] to extract features from bi-phasic hyperspectral images. Hao et al. [4] proposed a method that applied the fuzzy c-mean clustering algorithm and Markov random field in Change Vector Analysis (CVA) [3,5] for analyzing bi-phasic hyperspectral images. It generates the initial change map and calculates the probability of belonging to specific clusters. At last, it obtains the change detection map. Li et al. [6] proposed a feature selection method for hyperspectral remote sensing images based on the HSIAO framework, which optimizes band selection using the Jeffries–Matusita distance and evolutionary algorithms, significantly improving the efficiency and accuracy of downstream classification tasks.

With the progress in machine learning and its emergence in applications, conventional machine learning methods have been exploited in remote change detection. Touazi et al. [7] regarded changed and unchanged detection as a context-independent binary classification problem and proposed a scheme based on K-Nearest Neighbor (k-NN) to update the change detection decision from the feedforward neural network. Nemmour et al. [8] used a support vector machine (SVM) in the application of mapping urban extensions. Volpi et al. [9] implemented supervised change detection by reading the contextual information with support vector machines, which collected spatial and contextual information from a local texture distribution. Liu et al. [10] proposed difference representation learning with the application of stacked constrained Boltzmann machines for change detection in remote sensing pictures. This method analyzes the different images and detects changes between pixel pairs of multi-temporal remote sensing images.

In recent years, some change detection algorithms have been improved, and deep learning methods are becoming increasingly popular. Hyperspectral images gradually become mainstream data sources. Some classical deep learning frameworks applied to change detection are Convolutional Neural Network (CNN) [11], Long Short-Term Memory (LSTM) [12], Recurrent Neural Network (RNN) [13], etc. Du et al. [14] proposed an Unsupervised Deep Slow Feature Analysis (DSFA) network for multi-temporal hyperspectral image change detection, which effectively utilizes the excellent performance of deep networks in the fields of feature extraction and projection. Li et al. [15] proposed an unsupervised end-to-end framework based on deep learning for hyperspectral image change detection. Wang et al. [16] proposed a Siamese CNN with Spectral–Spatial-Wise Attention (SSA-SiamNet) for hyperspectral image change detection. This method affords more attention to informative channels and locations to improve spectral and spatial features adaptively. To deal with the inability of the existing methods to handle the details of complex objects at different scales in hyperspectral images, Yang et al. [17] proposed a deep multi-scale pyramidal network with spatial–spectral residual attention enhancement for hyperspectral image change detection. The network was employed to effectively utilize the excellent performance of deep networks in feature extraction and projection.

Transformer structure models, with their attention mechanism, have shown their capability of capturing complex features in the field of natural language processing. In recent years, this model has also been introduced into image detection tasks to help improve CNNs in identifying long-range dependencies. The transformer model has a self-attention module, which is effective at memorizing global information when processing an image. This is particularly important for recognizing changing regions in an image. Thus, the transformer model can effectively capture subtle differences between two images, even if these differences are spatially far apart. For example, the Position–Time-Aware Transformer (PT-Former) significantly improves change detection accuracy by modeling the position and time relationships in dual-temporal images. In addition, the transformer model performs better in handling problems such as pseudo-changes and incomplete edges when dealing with multi-temporal remote sensing images. This is because the transformer model can model positional and temporal correlations in dual-temporal RS images. The transformer model shows impressive potential in change detection tasks by aggregating contextual information of ground objects through fusion blocks and reconstructing spatial relationships guided by dual-temporal features. Fang et al. [18] proposed a deformable convolution-enhanced hierarchical transformer model for hyperspectral image classification (HSIC), known as SClusterFormer. This model incorporates a unique clustering attention mechanism that enhances the representation of homogeneous local details in 3D hyperspectral images (HSIs) and 2D morphological data while improving the discrimination of non-local structures. Zhang et al. [19] designed SwinSUNet using a transformer module based on the shift window mechanism. ChangeFormer [20] applies a hierarchical dual transformer encoder to create a model that efficiently acquires multi-scale remote sensing details. BIT-CD [21] utilizes ResNet for feature extraction and enhances the features extracted with an additional transformer module. Wang et al. [22] proposed a framework, UVACD, for the detection of dual-temporal image changes, which combines a CNN and a visual transformer. MSTDSNet-CD [23] introduced deep supervision in a CD network constructed with a CNN and a Swin transformer module for the detection of urban area changes. ICIF-Net [24] extracts semantic features of images of the dual-temporal phase in parallel. This method then feeds these features into a cross-scale fusion module, which provides integrated global details for change detection of remote sensing image pairs.

In various CV tasks like image classification and semantic segmentation, multi-scale feature extraction is able to capture the information of an image at both the local scale and global scale, thus improving the performance and generalization of the model. In recent years, multi-scale feature extraction techniques have been widely used and rapidly developed in several fields. Lei et al. [25] proposed a CNN module known as pyramid pooling. The module consists of three different sizes of convolutional kernels, in which each kernel takes charge of features within a certain scale range; thus, the model can observe objects of different sizes in images. SLDDNet [26] introduces a central aggregation strategy, fusing global semantic information with local detailed features. MSCCA-Net first introduces a bottom-up hierarchical fusion strategy and optimizes the feature fusion process by using the multi-scale cascade cross-attention (MSCCA) mechanism, which redistributes the spatial and semantic information in images. In this way, the accuracy of detecting small targets is significantly improved. Thus, this method outperforms the other existing techniques regarding comprehensive performance.

Although CNNs have achieved remarkable results, the existing methods are still deficient in dealing with the fusion of global and local feature information as well as the mining of deep semantic information. Concerning these issues, this paper proposes an innovative CNN-based multi-scale cross-attention network. The following points summarize our research contributions:

(1) Multi-scale feature extraction mechanism: We introduce a multi-scale feature extraction mechanism, which achieves an effective fusion of image information among multiple scales; thus, the model’s effectiveness in capturing image details is enhanced.
(2) Cross-attention module: This module improves the model’s ability to understand semantic information. In this way, the model can better recognize the key change parts of the image.
(3) Experimental validation: Our experiments are conducted on three public datasets. The results show that the algorithm proposed in this paper outperforms some current representative change detection algorithms in several key performance metrics, which validates the effectiveness of our method.

## 2. Methods

### 2.1. Network Structure

**Figure 1:**
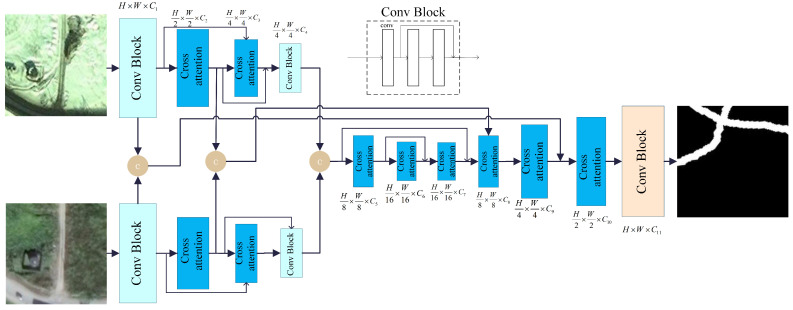
MSCAnetwork structure.

The overall structure of MSCA is shown in Figure 1, a cross-attention hierarchical network. The architecture processes dual-temporal remote sensing data with three input channels and generates two-channel change detection maps. A weight-shared two-path encoder progressively downsamples the input bitemporal remote sensing imagery through hierarchical compression. The downsampling process is realized through a patch merging layer. After the feature maps are downsampled to half the size of the original image, they are input into the semantic map generation module in the cross-attention module, which generates low-spatial-resolution semantic maps through nonlinear projection. These semantic maps are computed with the feature images for cross-attention. When the spatial resolution is reduced to 1/4 of the original image, the encoder concatenates the feature maps extracted from its dual-branch part, and the number of channels is modified for dual-branch feature fusion. The fused feature map passes through two encoding stages and then enters the decoding stage, where the change information is gradually recovered by the cross-attention and convolution modules. Finally, the change map is recovered to the same spatial resolution as the input image, and the number of channels is adjusted to two by the convolution module to output the final change map. The working procedure of MSCA initially extracts low-level features from remote-sensing images via the convolution module, followed by downsampling to reduce the spatial resolution and computational burden of the CS module. The semantic map is generated by nonlinear projection and represents a highly discriminative feature matrix. The spatial resolution of the semantic map is adjusted to half of the minimum resolution level. This cuts down the self-attention’s complexity from O(n^2^) to O(n) yet still enables efficient global information aggregation and precise error refinement. The $C_{i}$ values in Figure 1 represent the channel values of different stage features and semantic maps. By adjusting the value of $C_{i}$, the model can deal with different channel change scales.

### 2.2. Semantic Generation Module

**Figure 2:**
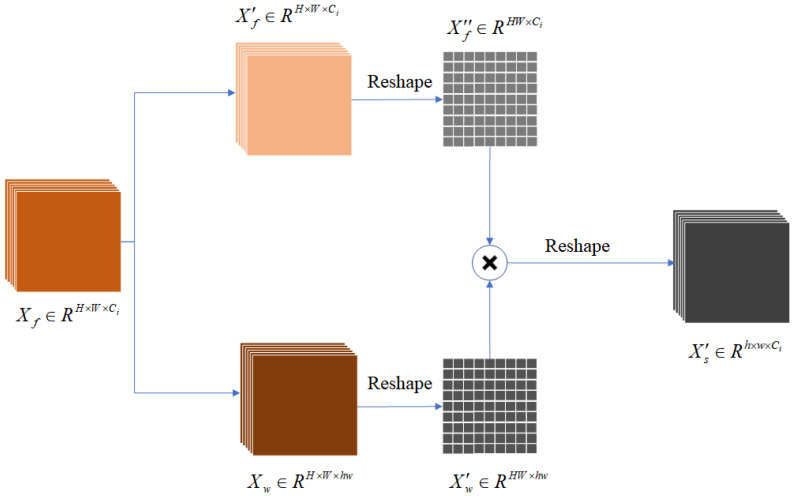
Semantic mapping generation module.

The CA module utilizes two matrices with distinct spatial resolutions as input data. Therefore, a semantic generation module is introduced in the CA module to create an initial semantic mapping. This module uses convolutional layers to extract features, followed by initial semantic mapping generation by linear mapping. The two mappings are the starting inputs of the CA module. Figure 2 presents the detailed structure characterizing the semantic generation module.

In this process, the features $X_{f}$ are first assigned to two different subspaces $X_{f}^{\prime }$ and $X_{w}$. This ensures that the spatial resolution of both subspaces remains consistent with the original features while adjusting the channel count of $X_{w}$ to match the product of the semantic map’s length and width. Next, the semantic mapping of $X_{w}$ is reshaped and processed by the softmax function. The processed $X_{w}$ becomes a weight matrix when operating with the reshaped $X_{f}$ matrix. This step resigns the weights of information in $X_{f}$, and generates a low-resolution semantic map that facilitates information aggregation.

### 2.3. Cross-Attention

**Figure 3:**
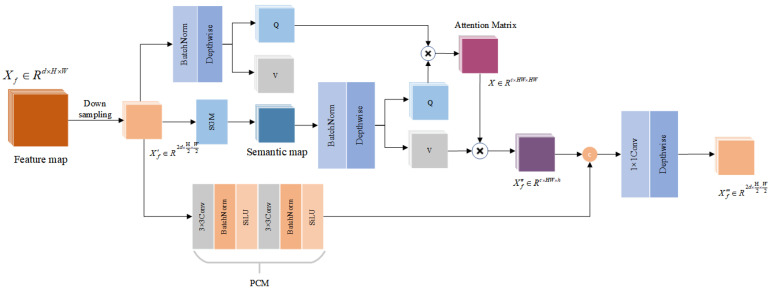
Cross-attention network structure.

The main structure of cross-attention is shown in Figure 3, which is based on the transformer structure and built on multi-head attention (MHSA) and Fully Connected Layer (FFN). In vision tasks, in order to comply with the transformer, each feature map input is flattened from $X_{f}\in R^{H\times W\times d}$ to $X_{f}\in R^{n\times d}$. H is the height of the feature map, W is the width of the feature map, and d is the dimension of the feature map, n=H×W. In order to compute the attention of $X_{t}$, it is projected to the query vector query $Q\in R^{n\times d}$, the key vector key $K\in R^{n\times d}$, and the value vector value $V\in R^{n\times d}$. The self-attention equation is calculated as below:

$$Attention\left(Q,K,V\right)=softmax\left(\frac{QK^{T}}{\sqrt{d}}\right)V$$

Here, $QK^{T}$ denotes the dot product of the transpose of the query matrix *Q* and the key matrix *K*. $\sqrt{d}$ is a scaling factor to control the scale of the dot product, which prevents the gradient from disappearing before the softmax calculation when the dot product is too large.

The multi-head attention mechanism is the core component of the transformer model, which learns information from different representation subspaces. It has multiple attention heads working in parallel to capture dependencies of different positions in the sequence and thus learn information from different representation subspaces to enhance its expression ability. The formula of multi-head attention is as follows:

$$MHSA\left(Q,K,V\right)=Concat\left(head_{1},\ldots ,head_{n}\right)W^{O}$$

$$head_{i}=Attention\left(QW_{i}^{Q},KW_{i}^{K},VW_{i}^{V}\right)$$

$W_{i}^{Q}\in R^{d_{m}\times d_{w}}$, $W_{i}^{K}\in R^{d_{m}\times d_{k}}$, $W_{i}^{V}\in R^{d_{m}\times d_{v}}$ are learnable parameters. $d_{K}$ and $d_{V}$ are the hidden dimensions of the projected subspace. $d_{m}$ is the embedding dimension. In the cross-attention module, the feature extraction and representation capabilities are improved by enhancing the interaction between the feature graph and the semantic graph, thus capturing their cross-module relationships. The idea is to optimize the performance of downstream tasks by focusing on critical regions in the feature graph and semantic graph while suppressing irrelevant information through self-attention and cross-attention mechanisms. The input to the module is the feature map, which is downsampled and input to the semantic generation module to generate semantic vectors. The feature graph generates query vectors and value vectors through depth-separable convolution operation, respectively; the semantic graph generates query vectors and value vectors in the same way. Subsequently, these query vectors and value vectors are rearranged, and the attention weights between the feature graph and the semantic graph are computed using the self-attention mechanism. The process adjusts the attention computation by a scaling factor to ensure the stability of the computation. The specific calculation formula is as follows:

$$Cross-Attention\left(Q,\bar{K},\bar{V}\right)=softmax\left(\frac{Q{\bar{K}}^{T}}{\sqrt{d}}\right)\bar{V}$$

The resulting attention graph is used for weighted merged feature and semantic graph representations, and the cross-attention scores of the feature and semantic graphs are normalized by the softmax function to generate a weighted feature representation. To further improve the stability of the model, a dropout layer is added to the cross-attention part to avoid overfitting. The output of the feature map is further optimized by the PCM module, which is jump-connected to the feature map weighted by the attention matrix, which extracts more stable and robust features through a series of convolutional layers, batch normalization, and activation functions to further improve the representation of the model. The final output of the feature map is projected to the desired output dimension by generating the corresponding output features through convolutional layers. The whole process, through the combination of the two-way attention mechanism and the positional conditioning module, captures the correlation between regions and improves the model’s performance in tasks such as image segmentation and target detection.

### 2.4. Loss Function

Change detection (CD) task requires delicate predictions for each pixel. To compute the loss during the training process, we used the weighted cross-entropy (WCE) loss function [27] and the Dice loss [28] function. The cross-entropy (CE) loss function was introduced to address the problem of sample imbalance in the change and non-change domains. We model the predicted change map $\hat{Y}$, which is a set containing H×W pixel points, where H and W represent the height and width of the image. These pixel points constitute the predicted change scenarios for subsequent loss calculations.

The WCE loss function can be defined in the following form:

$$L_{wce}=\frac{1}{H\times W}\sum_{i=1}^{H\times W}weight\left[class\right]\cdot \left(\log\left(\frac{e^{{\hat{y}}_{i}\left[class\right]}}{e^{(}{\hat{y}}_{i}\left[0\right]+{\hat{y}}_{i}\left[1\right])}\right)\right)$$

${\hat{y}}_{i}\left[\mathrm{class}\right]$ belongs to 0, 1, representing change and non-change. The Dice coefficient describes the similarity of the set and takes values between [0, 1]. Y denotes the set of pixels of the true label, and $\hat{Y}$ denotes the set of pixels of the predicted map. The Dice coefficient is calculated as follows:

$$DiceCoefficient=\frac{2|Y\cap \hat{Y}|}{\left|Y|+\right|\hat{Y}|}$$

$|Y\cap \hat{Y}|$ represents the number of pixels that are predicted correctly. Dice coefficient is defined as below:

$$L_{dice}=1-DiceCoefficient$$

The smaller Dice loss is, the more similar the ground truth and predictions are. The loss in total is defined as follows:

$$L_{loss}=L_{wce}+L_{dice}$$

## 3. Experiments

### 3.1. Datasets

The three publicly available datasets used in this work cover diverse regions and resolutions, as detailed in Table 1. This includes urban and rural areas, different years, and both aerial and satellite imagery, making them suitable benchmarks for evaluating the robustness of change detection models.

1. CDD [29]: The CDD dataset, collected from Google Earth and consisting of 16,000 remote sensing image pairs, includes a variety of change scenarios ranging from synthetic images with no or minor relative object movement to real-world scenes exhibiting seasonal variations. The image size is 256 × 256. The dataset is divided into a training set containing 10,000 images and a validation set of 3000 images.
2. LEVIR-CD [30]: This dataset comes from 20 different areas of several cities in Texas, USA, which is a large remote sensing building change detection dataset, including houses, apartments, garages, and other types of buildings. The image size is 1024 × 1024.
3. SYSU-CD [31]: This dataset contains 20,000 pairs of 0.5 m aerial images with a size of 256 × 256, which were taken in Hong Kong between 2007 and 2014. The classes included in the dataset are recently built buildings, suburban expansion, changes in plantings, and road expansion.

### 3.2. Baselines

We have selected six different deep learning change detection methods for comparison as follows:

STANet [29]: This method captures the spatial–temporal relationship between images through a self-attention mechanism to extract more practical features. The network combines basic and pyramid-structured attention modules to handle changes at different scales.

ChangeFormer [20]: This method proposes a transformer-based dual network for remote sensing image change detection. It extracts multi-scale features through a hierarchical transformer encoder and fuses them through an MLP decoder to predict changes, achieving better performance than traditional CNN-based methods.

BIT-CD [21]: This method converts dual-temporal images into a small number of semantic tokens and models the spatial–temporal context by tokens using a transformer encoder. This method converts the context tokens back into pixel space via a transformer decoder to refine the features. This method improves prediction accuracy while keeping efficiency.

HANet [32]: This method effectively integrates multi-scale features and refines detailed features through a progressive foreground-balanced sampling strategy and a lightweight self-attention mechanism, HAN module. This method adopts a hybrid loss function to successfully alleviate the data imbalance problem, which is validated on two extremely imbalanced datasets.

SNUNet [27]: This method enhances the accuracy of change detection by employing a densely connected Siamese network architecture, which mitigates the loss of localization information in deep networks through compact information transfer between the encoders and decoders, as well as between decoders themselves. Furthermore, it introduces an Ensemble Channel Attention Module (ECAM) for deep supervision. The ECAM allows for the refinement of the most representative features from different semantic levels, which are then utilized for the final classification.

SEIFNet [33]: This method proposes a spatiotemporal enhancement and interlevel fusion network (SEIFNet) for remote sensing image change detection. It enhances the representation of changing objects by capturing global and local information from bitemporal feature maps using a spatiotemporal difference enhancement module (ST-DEM). Additionally, SEIFNet integrates interlevel features through an adaptive context fusion module (ACFM) and refines the change detection results with a progressive decoder. This approach effectively highlights changed regions while suppressing noise interference and improving boundary details.

### 3.3. Metrics

In order to quantitatively identify the performance of models, we select the metrics that are mainstream in change detection tasks, including precision, recall, OA, IoU, and F1. The formulas for these metrics are shown below:

$$\mathrm{precision}=\frac{\mathrm{TP}}{\mathrm{TP}+\mathrm{FP}}$$

$$\mathrm{recall}=\frac{\mathrm{TP}}{\mathrm{TP}+\mathrm{FN}}$$

$$\mathrm{OA}=\frac{\mathrm{TP}+\mathrm{TN}}{\mathrm{TP}+\mathrm{FN}+\mathrm{TN}+\mathrm{FP}}$$

$$\mathrm{IoU}=\frac{\mathrm{TP}}{\mathrm{TP}+\mathrm{FP}+\mathrm{FN}}$$

$$F1=2\times \frac{\mathrm{precision}\times \mathrm{recall}}{\mathrm{precision}+\mathrm{recall}}$$

### 3.4. Comparison Experiments

#### 3.4.1. Results Analysis on CDD Datasets

**Figure 4:**
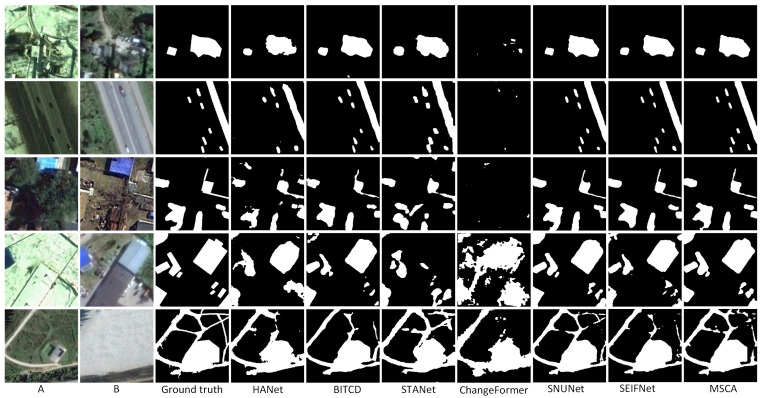
Prediction results of all methods on CDD datasets. (A refers to T1 image, B refers to T2 image).

As shown in Table 2, MSCA performs well in all evaluation metrics. Specifically, MSCA achieves the highest values in precision (96.39), F1-score (96.19), and IoU (92.67). The overall accuracy (OA) also achieves a high value of 99.10, which demonstrates that MSCA reduces the false prediction rate while accurately identifying the change regions. MSCA achieves a better balance between precision and recall than baseline methods such as ChangeFormer and BIT-CD. The superior performance of MSCA is mainly attributed to its advanced multi-scale feature extraction mechanism and cross-attention module. This design enables MSCA to capture global and local contextual information effectively, so MSCA is particularly outstanding in dealing with scenes with complex spatial structures and small changes. The performance of traditional methods, such as HANet and STANet, is inferior, e.g., the F1-score and IoU of STANet are 91.56 and 84.44. The IoU of HANet is even lower, only 79.19. In Figure 4, compared with ground truth, the change detection results output by MSCA are highly consistent in boundary details and overall integrity and can accurately describe the shape and edges of the changed area. In contrast, other models like HANet and STANet have obvious omissions in complex scenes. BIT-CD has incomplete segmentation dealing with complex change regions, although it performs well in some details. Especially in detecting small targets and delicate changes, MSCA, with its multi-scale feature extraction and cross-attention mechanism, shows excellent robustness and generalization ability. The detection results are almost the same as those of ground truth in both small-scale details and large-scale structural changes, further proving the technical advantages of MSCA in remote sensing image change detection.

#### 3.4.2. Results Analysis on LEVIR-CD Datasets

**Figure 5:**
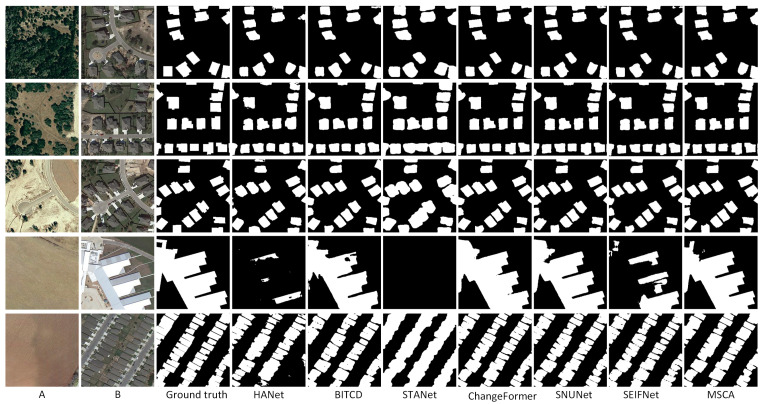
Prediction results of all methods on LEVIE-CD datasets.(A refers to T1 image, B refers to T2 image).

On the LEVIR-CD dataset, MSCA also demonstrates strong detection capability. As shown in Table 3, it achieves an F1-score of 91.02, which is the most outstanding among all experiments. Other metrics are also close to the optimal level, and it has an advantage in overall detection balance. The performance of MSCA indicates its ability to effectively handle complex urban and infrastructure change detection tasks. Figure 5 shows the detection results of the different methods on the LEVIR-CD dataset. HANet and STANet have remarkable errors on some images. In the fourth row of building change detection, HANet and STANet omitted huge areas. STANet is more ambiguous in the boundary processing, and the detection results of some areas are missing. In contrast, MSCA can accurately segment the change region, and its boundary integrity is highly consistent with ground truth.

#### 3.4.3. Results Analysis on SYSU-CD Datasets

**Figure 6:**
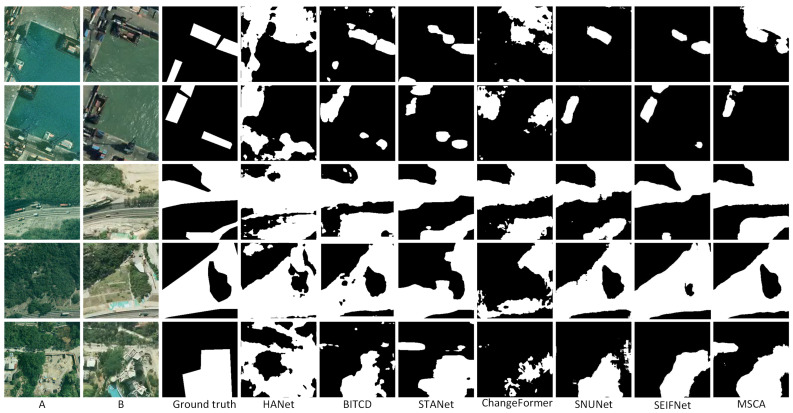
Prediction results of all methods on SYSU-CD. (A refers to T1 image, B refers to T2 image).

The change scenarios are more complex in the SYSU-CD dataset. Nevertheless, MSCA achieves the highest F1-score (80.76) and IoU (67.72), as shown in Table 4. MSCA’s significant performance improvement is due to the effective integration of its cross-attention mechanism. This mechanism effectively alleviates the problem that other methods fall into. For example, STANet tends to ignore changes on a small scale. The F1-score of STANet is only 78.12, and the IoU is only 64.10, which is relatively weak. In Figure 6, the change detection results of the models on the SYSU-CD dataset show large differences. In some complex scenarios (e.g., the second and third rows), MSCA can accurately segment the change regions, whereas the other methods have obvious false positives in these scenarios. The results of HANet especially have more background noise, while STANet has a blurring phenomenon in boundary processing.

### 3.5. Ablation Study

To validate the effectiveness of the proposed cross-attention (CA) module, we conduct a series of ablation experiments. Specifically, we compare the complete model with two variants: one in which the CA module is replaced by standard convolution layers and another baseline version without both the semantic perception and cross-attention modules.

The quantitative results are presented in Table 5, evaluated on three benchmark datasets: LEVIR-CD, CDD, and SYSU-CD. From the results, it can be observed that the integration of the CA module consistently improves the F1-score and IoU across all datasets. On the CDD dataset, the F1-score increases from 95.28% to 96.19%, and IoU rises from 91.00% to 92.67%, demonstrating that CA can better capture fine-grained and structural changes. On the more challenging SYSU-CD dataset, the CA module brings a significant boost in recall (from 74.87% to 78.95%) and improves the overall accuracy slightly. Notably, the improvements on the CDD and SYSU-CD datasets highlight the CA’s effectiveness in modeling complex change scenarios.

**Figure 7:**
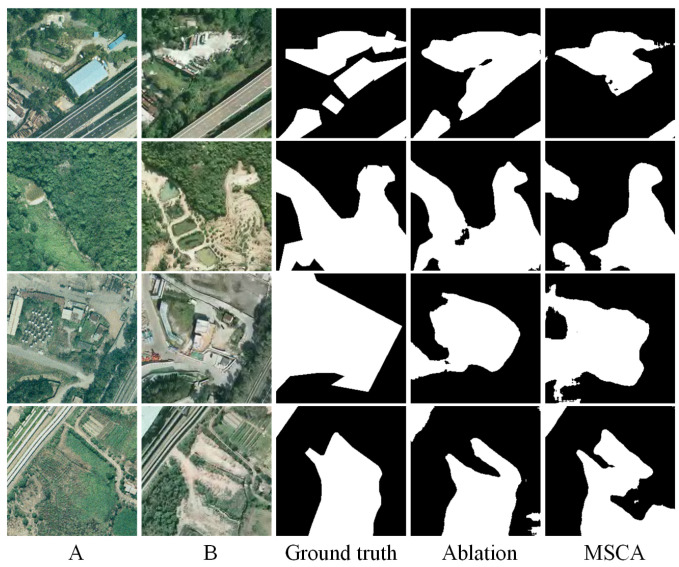
Ablation experiments on the SYSU-CD dataset. (A refers to T1 image, B refers to T2 image).

**Figure 8:**
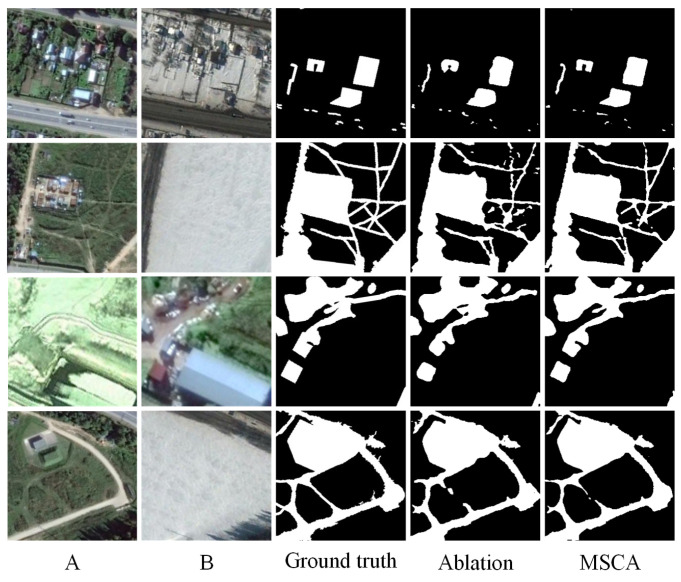
Ablation experiments on the CDD dataset. (A refers to T1 image, B refers to T2 image).

**Figure 9:**
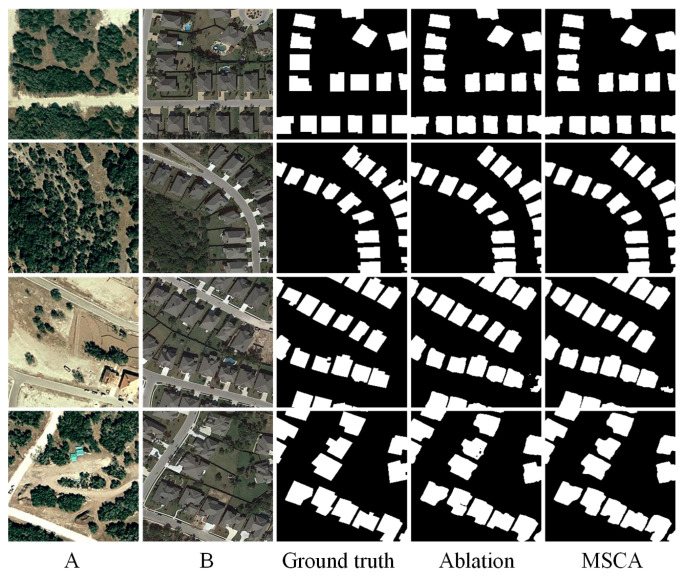
Ablation experiments on the LEVIR-CD dataset. (A refers to T1 image, B refers to T2 image).

To more intuitively demonstrate the impact of the CA module, we also provide visual comparisons of the ablation experiments, as shown in Figure 7, Figure 8 and Figure 9. For clarity, we present these results separately. Each figure includes input images A and B, the ground truth change map, the output of the ablation model, and the output of the full model with MSCA. From these visualizations, it is evident that the CA-enhanced model produces more accurate and cleaner change maps, particularly in preserving object boundaries, suppressing false positives, and detecting fine-grained structural changes. In contrast, the ablation variant often suffers from noise, missed detections, or fragmented regions—especially in challenging scenes with complex textures or subtle changes. This highlights the importance of both semantic feature extraction and the cross-attention mechanism in change detection tasks.

In summary, the ablation study demonstrates that the proposed CA module significantly improves the model’s discriminative power and overall accuracy, proving its practical value in enhancing change detection performance.

### 3.6. Impact of Input Noise and Model Robustness

**Figure 10:**
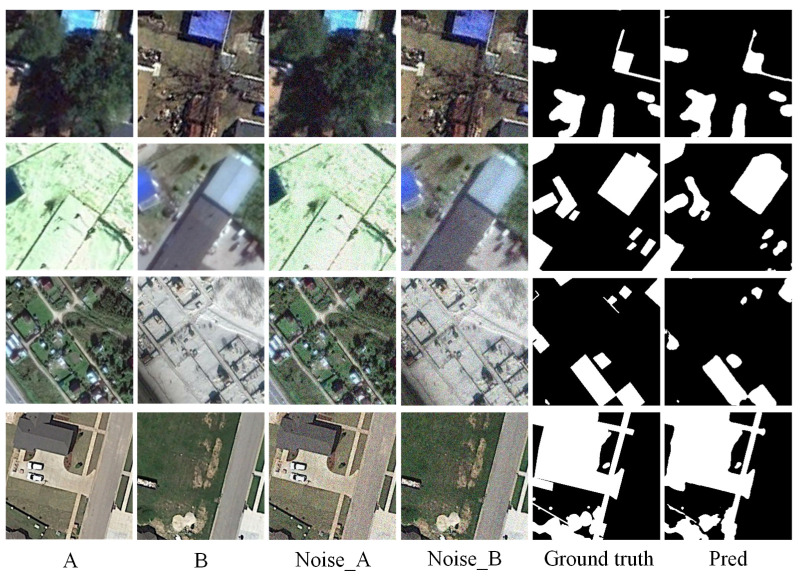
Experiment results on CDD dataset with Gaussian noise (Noise_A, Noise_B). (A refers to T1 image, B refers to T2 image).

**Figure 11:**
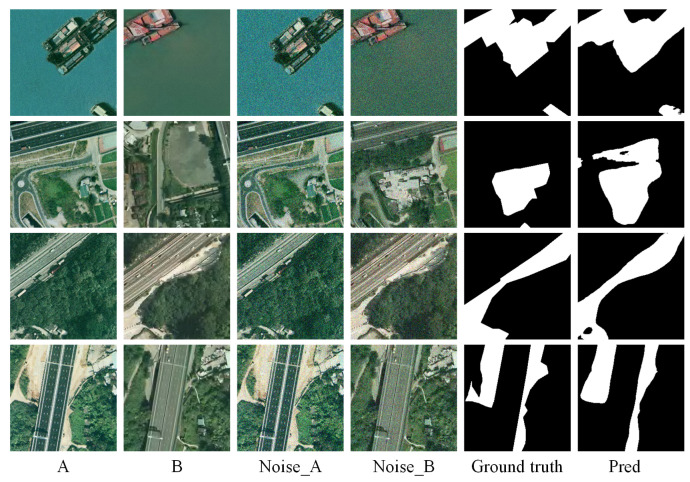
Experiment results on SYSU-CD dataset with Gaussian noise (Noise_A, Noise_B). (A refers to T1 image, B refers to T2 image).

To evaluate the robustness of the proposed MSCA framework under noisy conditions, we conduct experiments by adding Gaussian noise (σ = 0.1) to the input images. We carry out experiments on two representative datasets: CDD, which covers a wide range of seasonal and structural changes, and SYSU-CD, which contains diverse urban development scenarios with fine-grained annotations. Quantitative results are shown in Table 6, and visual comparisons are presented in Figure 10 and Figure 11.

Table 6 shows that MSCA maintains high performance even under noisy conditions. For instance, when Gaussian noise is added to the CDD dataset (denoted as CDD+gn), the F1-score only drops slightly from 96.19% to 95.98%, and the OA remains above 99%. On the SYSU-CD dataset, the F1-score decreases marginally from 80.76% to 80.03% after noise perturbation, confirming the model’s resilience.

Figure 10 and Figure 11 visualize the results. Despite noise interference, the predicted change maps align closely with the ground truth, indicating that MSCA effectively captures essential semantic changes and suppresses irrelevant noise.

These results demonstrate that our model exhibits strong robustness against common image degradations, which is essential for real-world remote sensing applications where image quality may be compromised due to atmospheric or sensor-induced distortions.

### 3.7. Visualization and Interpretability of Cross-Attention Mechanism

**Figure 12:**
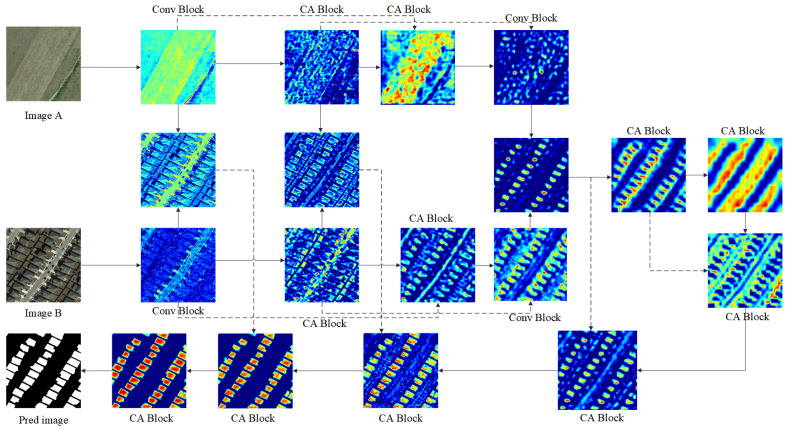
Network visualization taking bitemporal RS images from LEVIR-CD as an example. Dashed lines indicate skip connections.

To enhance the interpretability of attention mechanisms, particularly the cross-attention (CA) module, we provide a series of visualizations in the form of heatmaps (as shown in Figure 12). These heatmaps highlight the regions of the input images (Image A and Image B) that the model focuses on during different stages of the feature fusion and decoding process.

From the visualizations, it is evident that the CA blocks progressively enhance the attention to semantically meaningful structures such as roads, buildings, and edges. This attention is not random; rather, it corresponds to areas critical for accurate prediction, as shown in the final segmentation output. By visualizing intermediate features, we can better understand how the model integrates change information from bitemporal images.

These heatmaps serve as a valuable interpretability tool, offering insights into the decision-making process of the model. This is particularly important in high-stakes applications such as urban planning or disaster management, where understanding the model’s reasoning can build trust and guide further human-in-the-loop analysis.

### 3.8. Generalization and Overfitting Mitigation

Although the proposed MSCA framework achieves strong performance on three widely used benchmark datasets (CDD, LEVIR-CD, and SYSU-CD), concerns may arise regarding its ability to generalize to unseen domains due to potential dataset-specific overfitting. To address this, we adopt several strategies to enhance the model’s robustness and generalization capability:

1. Data Augmentation: To increase data diversity and reduce the risk of overfitting, we employ various data augmentation techniques during training, including random horizontal and vertical flips, rotations, color jittering, and scaling. These augmentations simulate different spatial arrangements and lighting conditions, helping the model to generalize beyond the training distribution.
2. Dropout Regularization: Dropout layers are incorporated within the attention modules to prevent the co-adaptation of neurons and reduce reliance on specific feature patterns. This regularization technique encourages the model to learn more robust, distributed representations, thereby improving its generalizability.
3. Semantic Abstraction in Cross-Attention: The semantic map projection embedded in the cross-attention module allows the model to operate on high-level abstractions rather than raw pixel differences. This semantic-level reasoning helps the model to identify meaningful changes across different domains and reduces the risk of overfitting to low-level, domain-specific noise.

Through this combination of regularization, augmentation, and architectural design, the MSCA model demonstrates strong potential for real-world deployment across varied and unseen remote sensing scenarios.

## 4. Conclusions

In order to enhance the interaction between local and global features, this paper proposes a CNN-based multi-scale cross-attention network (MSCANet) specifically for remote sensing image change detection (DC). The model incorporates the concept of serial–parallel correlation. The CA module handles global feature extraction, while the PCM module focuses on local feature capture to supplement the detailed information that may be lost due to aggregation. The MSCANet employs an intermediate-stage fusion strategy, distinguished from the traditional early or late fusion methods. This strategy effectively fuses global and local information and enhances the model’s understanding of image semantics. We conducted experiments on three publicly available datasets: CDD, LEVIR-CD, and SYSU-CD. The experimental results show that our method outperforms the traditional methods. Specifically, MSCANet achieved an F1-score of 96.19% and IoU of 92.67% on the CDD dataset, 91.02% F1-score on LEVIR-CD, and 80.76% F1-score on SYSU-CD, demonstrating its strong capability in handling complex change scenarios. Although MSCANet has demonstrated strong robustness, the large number of parameters may still limit its application in resource-constrained environments. Future research could focus on optimizing the network architecture, reducing the unnecessary computational burden, and exploring more efficient model compression techniques. More practical change detection solutions may be deployed by reducing computational costs and storage requirements while ensuring detection accuracy.

## Figures and Tables

**Table 1:** Summary of the dataset characteristics.

Dataset	Resolution (m/pixel)	Patch Size (pixels)	Data Source	Region
CDD [29]	∼0.5–1.0	256 × 256	Google Earth imagery	Global urban/rural
LEVIR-CD [30]	0.5	1024 × 1024	Google Earth (Texas, USA)	20 urban areas in Texas
SYSU-CD [31]	0.5	256 × 256	Aerial imagery (HK Gov.)	Hong Kong (2007–2014)

**Table 2:** Detection results on CDD datasets.

Model	Pre	Rec	F1	Iou	OA
SNUNet	95.62	95.24	95.42	90.37	99.10
STANet	88.97	94.31	91.56	84.44	87.95
ChangeFormer	95.27	93.82	94.54	89.65	98.72
BIT-CD	95.86	94.59	95.00	90.88	98.88
HANet	92.44	84.68	88.39	79.19	97.12
SEIFNet	95.85	92.62	94.20	89.04	98.66
MSCA	96.39	95.99	96.19	92.67	99.10

**Table 3:** Detection results on LEVIR-CD datasets.

Model	Pre	Rec	F1	Iou	OA
SNUNet	89.18	87.17	88.16	78.83	98.82
STANet	80.99	91.92	85.79	75.12	98.46
ChangeFormer	91.53	88.86	90.17	82.10	99.01
BIT-CD	90.95	88.57	90.23	82.19	99.02
HANet	89.56	87.13	88.33	79.10	98.83
SEIFNet	89.67	85.87	89.14	80.41	98.93
MSCA	90.65	87.46	91.02	80.22	98.90

**Table 4:** Detection results on SYSU-CD datasets.

Model	Pre	Rec	F1	Iou	OA
SNUNet	74.55	65.15	67.68	61.11	88.78
STANet	82.36	74.30	78.12	64.10	90.18
ChangeFormer	84.99	70.93	77.33	63.04	90.19
BIT-CD	79.04	76.71	77.86	63.75	89.71
HANet	78.71	76.14	77.41	63.14	89.52
SEIFNet	79.47	78.28	78.87	65.11	90.10
MSCA	82.65	78.95	80.76	67.72	91.13

**Table 5:** Performance comparison of baseline and MSCA on three datasets.

Dataset	Method	Pre (%)	Rec (%)	F1 (%)	IoU (%)	OA (%)
Levir-CD	Baseline	92.45	88.42	90.39	82.47	99.04
	MSCA	90.65	87.46	91.02	80.22	98.90
CDD	Baseline	95.07	95.51	95.28	91.00	98.89
	MSCA	96.39	95.99	96.19	92.67	99.10
SYSU-CD	Baseline	85.02	74.87	79.62	66.15	90.96
	MSCA	82.65	78.95	80.76	67.72	91.13

**Table 6:** Experiment results of MSCA on two datasets with added Gaussian noise.

Method	Dataset	Pre (%)	Rec (%)	F1 (%)	Iou (%)	OA (%)
MSCA	CDD	96.39	95.99	96.19	92.67	99.10
	CDD+gn	96.35	95.61	95.98	92.27	99.05
	SYSU-CD	82.65	78.95	80.76	67.72	91.13
	SYSU-CD+gn	86.72	74.29	80.03	66.72	91.26

## Data Availability

The datasets generated during and analysed during the current study are available from the corresponding author on reasonable request.
